# Circulating tumour cells and PD-L1-positive small extracellular vesicles: the liquid biopsy combination for prognostic information in patients with metastatic non-small cell lung cancer

**DOI:** 10.1038/s41416-023-02491-9

**Published:** 2023-11-16

**Authors:** Zahra Eslami-S, Luis Enrique Cortés-Hernández, Léa Sinoquet, Ludovic Gauthier, Valentin Vautrot, Laure Cayrefourcq, Laure Avoscan, William Jacot, Stéphane Pouderoux, Marie Viala, Quentin Dominique Thomas, Pierre-Jean Lamy, Xavier Quantin, Jessica Gobbo, Catherine Alix-Panabières

**Affiliations:** 1 Laboratory of Rare Circulating Human Cells – University Medical Center of Montpellier, Montpellier, France; 2 CREEC/CANECEV, MIVEGEC (CREES), Université de Montpellier, CNRS, IRD, Montpellier, France; 3 European Liquid Biopsy Society (ELBS), Hamburg, Germany; 4 Department of Medical Oncology, Institut du Cancer de Montpellier, Montpellier University, Montpellier, France; 5 Biometrics Unit, Institut du Cancer de Montpellier, Montpellier University, Montpellier, France; 6 INSERM 1231, Label “Ligue National contre le Cancer “and Label d’Excellence LipSTIC, Dijon, France; 7 Department of Medical Oncology, Center Georges-François Leclerc, Dijon, France; 8 Agroécologie, Institut Agro Dijon, CNRS, INRAE, University Bourgogne Franche-Comté, Plateforme DImaCell, F-21000 Dijon, France; 9 Institut de Recherche en Cancérologie de Montpellier, INSERM U1194, Montpellier University, Montpellier, France; 10 Biopathologie et Génétique des Cancers, Institute d’Analyse Médicale Imagenome, Inovie, Montpellier, France; 11 Unité de recherche clinique, clinique Beau soleil, Montpellier, France; 12 Faculty of Medicine, University of Burgundy-Franche-Comté, Dijon, France; 13 Inserm, CIC1432, Module plurithématique, U2P, Dijon, France

**Keywords:** Non-small-cell lung cancer, Non-small-cell lung cancer

## Abstract

**Background:**

Circulating tumour cells (CTCs), circulating tumour DNA (ctDNA), and extracellular vesicles (EVs) are minimally invasive liquid biopsy biomarkers. This study investigated whether they predict prognosis, alone or in combination, in heterogenous unbiased non-small cell lung cancer (NSCLC) patients.

**Methods:**

Plasma samples of 54 advanced NSCLC patients from a prospective clinical trial. CtDNA mutations were identified using the UltraSEEK™ Lung Panel (MassARRAY® technology). PD-L1 expression was assessed in small EVs (sEVs) using an enzyme-linked immunosorbent assay.

**Results:**

At least one ctDNA mutation was detected in 37% of patients. Mutations were not correlated with overall survival (OS) (HR = 1.1, 95% CI = 0.55; 1.83, *P* = 0.980) and progression-free survival (PFS) (HR = 1.00, 95% CI = 0.57–1.76, *P* = 0.991). High PD-L1^+^ sEV concentration was correlated with OS (HR = 1.14, 95% CI = 1.03–1.26, *P* = 0.016), but not with PFS (HR = 1.08, 95% CI = 0.99–1.18, *P* = 0.095). The interaction analysis suggested that PD-L1^+^ sEV correlation with PFS changed in function of CTC presence/absence (*P* interaction = 0.036). The combination analysis highlighted worse prognosis for patients with CTCs and high PD-L1^+^ sEV concentration (HR = 7.65, 95% CI = 3.11–18.83, *P* < 0.001). The mutational statuses of ctDNA and tumour tissue were significantly correlated (*P* = 0.0001).

**Conclusion:**

CTCs and high PD-L1^+^ sEV concentration correlated with PFS and OS, but not ctDNA mutations. Their combined analysis may help to identify patients with worse OS.

**Trial registration:**

NCT02866149, Registered 01 June 2015, https://clinicaltrials.gov/ct2/show/study/NCT02866149.

## Background

Lung cancer is the second most frequent malignancy and the most common cause of cancer death in both sexes worldwide [1]. Non-small cell lung cancer (NSCLC) accounts for 80–90% of lung cancers, whereas small cell lung cancer has been declining in many countries in the last two decades. Adenocarcinoma, squamous-cell carcinoma and large-cell carcinoma are the most common NSCLC types. As most treatments are based on the information gained from the tissue biopsy at diagnosis, obtaining a precise description of the tumour using less invasive methods could improve screening and early detection and contribute to therapeutic decision-making.

In the cancer context, liquid biopsy defines a variety of screening approaches based on samples obtained in a minimally invasive manner. Currently, circulating tumour cells (CTCs) and circulating tumour DNA (ctDNA) are two of the most studied liquid biopsy analytes [2]. Recent studies indicate that also small extracellular vesicles (sEVs; also known as exosomes, size <200 nm) isolated from blood samples can correlate with tumour features [3, 4]. These analytes are particularly relevant in lung cancer because tumour biopsies are often difficult to obtain [5]. For example, in NSCLC, ctDNA can be used to detect targetable driver mutations in epidermal growth factor receptor (EGFR) [6], and CTC number is associated with prognosis [7]. Recently, our group showed that the presence of CTCs, particularly of programmed cell death ligand 1-positive (PD-L1^+^) CTCs, in patients with NSCLC is a robust prognostic marker that is independent of treatment and molecular subtyping [8]. Therefore, each individual biomarker holds useful biological and clinical information, and the combination of these biomarkers can be used to obtain complementary information. For instance, PD-L1^+^ sEV potential was investigated in different cancers alone [4, 9–14], and its combination with other biomarkers (e.g. ctDNA) was used to improve the identification of EGFR mutations in lung cancer [15]. Furthermore, it has been shown that combining sEVs, RNA, and ctDNA increases the sensitivity of EGFR mutation detection in plasma samples from patients with NSCLC [15]. Therefore, the combination of liquid biopsy analytes can provide more precise information on NSCLC prognosis compared with a single marker [16]. However, the mentioned studies were performed on homogenous populations in terms of treatment (before or after treatment; treatment type), cancer stage, and molecular subtype. Thus, in the current study, we wanted to investigate the prognostic value of three liquid biopsy analytes (CTCs, sEVs, and ctDNA), alone and in combination, in an unbiased heterogeneous cohort of patients with advanced NSCLC, regardless of treatment, cancer subtype and stage, to determine whether the combination of these liquid biopsy biomarkers gives better prognostic information than each of them on its own.

## Methods

### Patients and blood samples

**Fig. 1:**
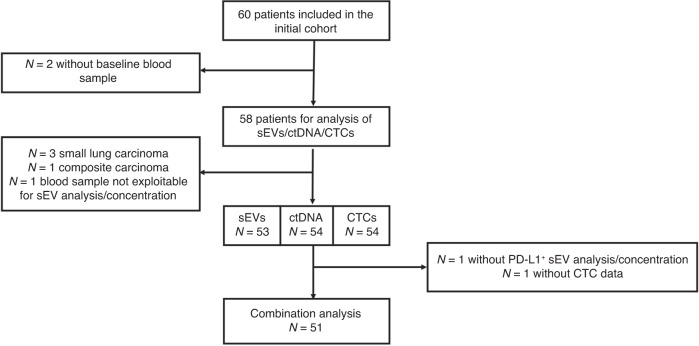
Flowchart of patients from the ALCINA 1 cohort with available data on the three liquid biopsy markers. sEVs small extracellular vesicles, ctDNA circulating tumour cells, CTCs circulating tumour cells, NSCLC non-small cell lung cancer.

Sixty patients (≥18 years of age) with histologically confirmed metastatic NSCLC (stage III and IV) were included in the prospective ALCINA 1 clinical trial (NCT02866149) from June 2016 to August 2018 to assess circulating biomarkers in different cancer types. The protocol was approved by the Montpellier University Hospital Centre ethics committee. This sample size of patients was employed to evaluate a technique’s viability and offer proof of concept, serving as the basis for more detailed investigations on a particular technique or indication. Blood sampling was performed at diagnosis, before the first treatment (*n* = 9), or later, at progression, before the next therapeutic line (*n* = 45). All patients gave their written informed consent. CTCs were analysed prospectively [8], whereas ctDNA and sEVs were evaluated retrospectively after all patients were included. Finally, only 51 patients had combined data on CTCs, sEVs and ctDNA (see flowchart in Fig. 1).

### CTC analysis

At inclusion, blood samples were collected in 10 ml CellSave® tubes (Menarini Silicon Biosystems ref: 7900005) and processed using the CellSearch® CTC kit (Menarini Silicon Biosystems ref. 7900001) according to the manufacturer’s instructions. Then, all samples were analysed at room temperature using the CellSearch® system (Menarini^©^ Silicon Biosystems). Briefly, the CellSearch® system includes a first step in which EpCAM^+^ CTCs are enriched and captured, followed by CTC detection using anti-cytokeratin-phycoerythrin and anti-CD45-allophycocyanin (to exclude leukocytes) antibodies and nuclear staining with 4′-6-diamidino-2-phenylindole. After immunocytochemical staining, immunomagnetically labelled cells are kept in a magnetic field and scanned using the CellTracks AnalyzerII® (Menarini^©^ Silicon Biosystems). The results of the CTC analysis in the 54 patients were reported previously [8] and are summarized in Supplementary Table 1.

### ctDNA analysis

#### Plasma isolation and ctDNA extraction

Plasma was isolated from 7.5 ml of blood drawn in EDTA tubes by centrifugation at 300 g for 10 min. The supernatant was then centrifuged at 1800 g for 10 min and at 15,000 g for 20 min to remove the remaining debris. Plasma aliquots of 1 ml were stored at −80 °C until ctDNA isolation with the Circulating Nucleic Acid kit (55114, Qiagen) according to the manufacturer’s protocol. ctDNA yield was determined with the Qubit™ 1× dsDNA HS Assay Kit (Q33230, Thermo Fisher Scientific).

#### Quantitative and qualitative analysis of ctDNA using the Liquid IQ® panel

Preanalytical parameters were assessed in a single reaction using 1.5 µL of ctDNA with the Liquid IQ® Panel and MALDI-TOF-based analysis with the MassARRAY® System (Agena Bioscience, San Diego, CA, USA). This method allows detecting long DNA templates originating from cell necrosis, white blood cell contamination, and amplifiable ctDNA copies to calculate the optimal ctDNA input.

#### ctDNA detection using the UltraSEEK^TM^ Lung panel

The UltraSEEK^TM^ Lung Panel was used to detect 74 different hot-spot mutations in five genes relevant to NSCLC: 46 mutations in *EGFR*, 15 mutations in Kirsten rat sarcoma virus *(KRAS)*, 4 mutations in B-Raf (*BRAF)*, 4 mutations in erb-b2 receptor tyrosine kinase 2 *(ERBB2)*, and 4 mutations in phosphatidylinositol-4,5-bisphosphate 3-kinase catalytic subunit alpha (*PIK3CA*). Multiplex PCR to target specific areas in the five genes was followed by variant-specific single base extension using chain terminators labelled with biotin. Then, the specific mutant allele was captured by streptavidin-activated magnetic beads, leading to an increased signal in the presence of the mutation.

#### UltraSEEK^TM^ data analysis

Data were analysed with the Typer software version 4.0.26.74 (Agena Bioscience, San Diego, CA, USA). The signal intensity for the mutant allele was normalized to the capture control peaks. An intensity value = 1 indicates that the peak intensity in the mutant allele is equal to the peak intensity of the average of the five capture control peaks. The capture control peaks are biotin-labelled, non-reactive oligonucleotides that are added to the extension reaction and used as an internal control for the streptavidin-bead capture and elution of the mutant extension product steps. Mutant allele calls were returned by an automated software report specific for the UltraSEEK™ Lung Panel. A signal-to-noise ratio ≥6 and a *z*-score ≥7 were considered significant. For allele calling, the reporter algorithm considered the instrument-specific baseline for each mutation assay. The assay-specific noise was assessed by analysing a cohort of wild-type samples and the mutant call significance was controlled by analysing commercial mutation controls as titration of the mutant allele frequencies down to the limit of detection of 0.1%.

### Analysis of EVs

#### Isolation and characterization of sEVs

Thawed plasma samples (collected at inclusion) were centrifuged at 2000 g, 4 °C, for 20 min and then at 10,000 g, 4 °C, for 20 min. 500 µL of supernatant was mixed with 1X PBS, and incubated with Total Exosome Precipitation Reagent (4484450, Thermo Fisher Scientific) for 10 min after homogenization, according to the manufacturer’s protocol. sEVs were pelleted by centrifugation at 10,000 g for 5 min, and the supernatant was centrifuged again at 10,000 g for 30 s before collection. sEV pellets were carefully resuspended in 60 µl of 1X PBS filtered through a 0.1 µm filter. An aliquot of 2.5 µL of this sEV solution was used for nanoparticle tracking analysis (NTA) to determine the sEV size and concentration with an NS300 instrument (Malvern Panalytical, Malvern, UK).

Isolated sEVs were then incubated with antibodies against universal sEV markers: CD9 (sc-13118, Santa Cruz Biotechnology), ALIX (NB100-65678, Novus Bio), TSG101 (sc-7964, Santa Cruz Biotechnology), CD63 (NBP2-4225, BioTechne), CD81 (sc166028, Santa Cruz Biotechnology), HSP70 (ADI-SPA-810, Enzo Life science), PD-L1 (sc-50298, Santa Cruz Biotechnology), and GRP94 as negative control (ADI-SPA-850, Enzo Life Sciences). Briefly, sEVs and the A549 cell line (Human lung carcinoma, ATCC) were lysed on ice with 1X Cell Lysis Buffer (9803, Cell Signaling Technologies) supplemented with 1X complete protease inhibitor cocktail (Merck ref:11697498001) for 20 min, followed by sonication with a Branson Digital Sonifier SFX 150 (Emerson) (continuous emission, 20% amplitude, “micro” mode) for 10 s. sEV lysates were separated on SDS/PAGE gels, and proteins were transferred onto polyvinylidene fluoride membranes (Amersham GE Healthcare Life Sciences) for western blot analysis. After transfer, membranes were blocked with 5% bovine serum albumin for 1 h and incubated at 4 °C with antibodies overnight. Following incubation with secondary antibodies (Jackson ImmunoResearch), immunoreactions were revealed using ECL detection reagents (34095, ThermoFisher Scientific) and the Chemidoc MP system. Images were analysed with the Image Lab software (Bio-Rad Laboratories).

For electron microscopy analysis, sEV pellets were resuspended in 50 µL 1X PBS, and an aliquot was diluted 10 times in sterile water. 10 µL of diluted sample was deposited on a formwar/carbon-coated copper effluved grid and left for 4 min. After adding a drop of UranyLess solution (Delta Microscopies) for 60 s for contrast staining, excess liquid was absorbed on filter paper. Images were acquired with a Hitachi 7800 electron microscope (Hitachi high technologies, Tokyo, Japan).

#### Determination of PD-L1^+^ sEV concentration from plasma samples

An enzyme-linked immunosorbent assay (DB7H10, ELISA Quantikine PD-L1/B7H1 Human/Cynomolgus Monkey, R&D Systems) was used to quantify PD-L1^+^ sEV lysates with 1X Cell Lysis Buffer (9803, Cell Signalling Technologies). Protein concentrations were determined using standard curves established with a linear function. The blank value (lysis solution alone) was subtracted from the sample value.

### Statistical analyses

Qualitative variables were described using frequencies and percentages, and continuous variables with means, medians, and interquartile range. Percentages were calculated relative to the total population, excluding missing data. The Chi-square and the Fischer’s exact test were used to compare qualitative variables and the Kruskal–Wallis test for quantitative variables.

For ctDNA and sEV analyses, the survival endpoints were progression-free survival (PFS) and overall survival (OS). In the ctDNA analysis, survival curves associated with the presence of ctDNA harbouring *EGFR* or *KRAS* mutations were obtained using the Kaplan–Meier method. The effect of each mutation was assessed using the univariable Cox model.

For sEV analysis, PD-L1^+^ sEV and total sEV concentrations were treated as continuous variables in univariable and multivariable Cox models. The linearity assumption was confirmed using martingale residuals and spline regressions. Interactions between PD-L1^+^ sEV concentration and CTC presence were analysed. For the multivariable analysis, the effects of PD-L1^+^ sEV and total sEV concentrations were adjusted for three variables that were pre-selected according to the current knowledge and literature: CTC presence, number of previous systemic treatment lines, and tumour histological type.

To assess the prognostic impact of biomarker combinations, a multivariable Cox model (*P*-value < 0.05) that included ctDNA, CTCs, PD-L1^+^ sEVs was fitted. The effect of each combination was then estimated using contrast coefficients. To facilitate the interpretation, PD-L1^+^ sEV concentrations were dichotomized (high and low) using cut-offs obtained using the maximally selected rank statistics for OS and PFS [17].

All statistical tests were bilateral and a *P*-value < 0.05 was considered significant. All statistical analyses were performed with STATA, v.16.0 and R, v. 4.1.2.

## Results

### Patients’ characteristics

Blood samples from 54 patients with stage III–IV NSCLC were used for this study (Fig. 1). The patients’ mean age was 64.5 ± 11.8 years, 31 were men and 23 were women. Most patients had metastases (94.4%), and adenocarcinoma was the most frequent histological type (72.2%). The median time from diagnosis to inclusion was 18 months. The median follow-up was 44.9 months (95% CI 33.0–52.4). The cohort was heterogeneous in terms of molecular subtypes, treatment status, and treatment type. Specifically, 83.3% of patients had undergone at least one treatment before inclusion: systemic therapy (68.5%), surgery (25.9%), radiotherapy (24.1%), and radio-chemotherapy (7.4%) (Table 1).

**Table 1:** Patients’ characteristics.

**Age**	
*N*	54
Mean (SD)	64.5 (11.8)
Median (Q1; Q3)	67.0 (54.0; 74.0)
Missing	0
**Sex**	
Men	31 (57.4%)
Women	23 (42.6%)
**Smoking**	
Never smoker	7 (13.5%)
Previous smoker	37 (71.2%)
Current smoker	8 (15.3%)
Missing	2
**BMI (kg/m²)**	
*N*	50
Mean (SD)	23.8 (4.2)
Median (Q1; Q3)	23.4 (20.6; 26.3)
Missing	4
**Medical history of disease**	
No	11 (20.4%)
Yes	43 (79.6%)
**Histological type**	
Adenocarcinoma	39 (72.2%)
Squamous cell carcinoma	10 (18.4%)
Large cell neuroendocrine carcinoma	2 (3.7%)
Adenosquamous carcinoma	1(1.9%)
Undifferentiated large cell carcinoma	1 (1.9%)
basaloid carcinoma	1 (1.9%)
**Metastases**	
No	3 (5.6%)
Yes	51 (94.4%)
**If yes: Number of metastatic sites**	
*N*	51
Mean (SD)	2.7 (1.5)
Median (Q1; Q3)	3.0 (1.0; 4.0)
Missing	0
**Previous treatment(s)**	
No	9 (16.7%)
Yes	45 (83.3%)
Radiotherapy or radio-chemotherapy	17 (31.5%)
Surgery	14 (29.5%)
Systemic treatment	37 (68.5%)
One line	23 (42.6%)
Two lines	7 (13 %)
Three line	6 (11.1%)
More than three lines	1 (1.9%)
**Any mutation in ctDNA**	
No mutation	34 (63.0%)
At least one mutation	20 (37.0%)
**KRAS mutation**	
No	46 (85.2%)
Yes	8 (14.8%)
**EGFR mutation**	
No	41 (75.9%)
Yes	13 (24.1%)
**sEVs, Mean size**	
*N*	53
Mean (SD)	148.2 (17.2)
Median (Q1 ; Q3)	148.5 (134.7; 158.6)
Missing	0
**sEVs, Mode size**	
N	53
Mean (SD)	120.3 (9.9)
Median (Q1 ; Q3)	120.0 (112.1; 128.3)
Missing	0
**sEV concentration (x10**^**9**^ **particle/ml)**	
*N*	53
Mean (SD)	2622.2 (1924.7)
Median (Q1; Q3)	2460.0 (884.0; 3702.0)
Missing	0
**PD-L1**^**+**^ **sEV concentration (pg/ml)**	
*N*	52
Mean (SD)	16.9 (14.5)
Median (Q1; Q3)	12.0 (6.6; 25.5)
Missing	1
**PD-L1**^**+**^ **sEVs**	
No	6 (11.5%)
Yes	46 (88.5%)
Missing	1

### Circulating tumour DNA

ctDNA mutation status could be analysed in all 54 patients. At least one hot-spot mutation in *EGFR*, *KRAS*, *BRAF*, *ERBB2* or *PIK3CA* was detected in the ctDNA of 20/54 patients (37%). *KRAS* and *EGFR* mutations were the most frequently detected: 8 (14.8%) and 13 (24.1%) patients, respectively. *PIK3CA* mutations were detected in two patients, whereas *BRAF* and *ERBB2* mutations were not found in the ctDNA of any patient.

**Fig. 2:**
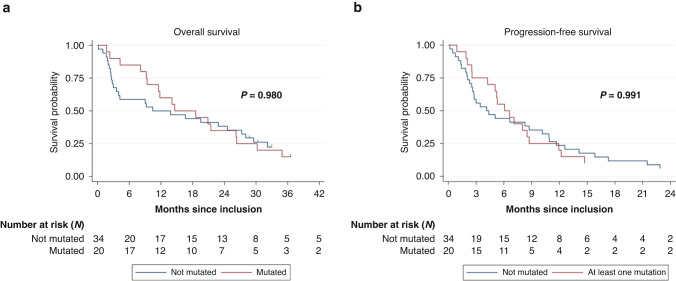
Correlation between presence/absence of mutations in ctDNA and overall survival and progression-free survival. **a** Kaplan-Meier curves for overall survival according to the presence/absence of *EGFR*, *KRAS*, *BRAF*, *ERBB2* or *PIK3CA* mutation(s) in ctDNA. **b** Kaplan-Meier curves for progression-free survival according to the presence/absence of ctDNA mutations.

Age, sex, smoking status, body mass index (BMI), medical history, histological type, tumour (T), nodes (N) and metastases (M) (TNM classification) and number of metastatic sites were comparable between patients with (*n* = 20) and without (*n* = 34) ctDNA mutations (Supplemental Table 2). Similarly, Kaplan–Meier analyses did not highlight any significant correlation between presence/absence of ctDNA mutations and OS (HR = 1.1, 95% CI = 0.55–1.83, *P* = 0.980) and PFS (HR = 1.0, 95% CI = 0.57–1.76, *P* = 0.991) (Fig. 2). The same analysis was then performed by dividing patients in function of the presence/absence of *KRAS* or *EFGR* mutations in ctDNA (Supplemental Fig. 1). The presence of *KRAS* or *EGFR* mutations also was not correlated with OS (HR = 1.41, 95% CI = 0.64–3.10, *P* = 0.407; and HR = 0.80, 95% CI = 0.41–1.59, *P* = 0.524) and PFS (HR = 1.52, 95% CI = 0.70–3.29, *P* = 0.308, and HR = 0.80, 95% CI = 0.43–1.51, *P* = 0.488, respectively).

The same analysis was done to evaluate the correlation between presence of mutations in cancer tissue samples and clinical outcomes. As observed for ctDNA, the presence of *KRAS* or *EGFR* mutations in cancer specimens was not correlated with OS (HR = 0.65, 95% CI = 0.32–1.31, *P* = 0.211; and HR = 0.54, 95% CI = 0.21–1.39, *P* = 0.168). *KRAS* mutations were not correlated with PFS (HR = 0.87, 95% CI = 0.46–1.65, *P* = 0.677), whereas *EGFR* mutations were significantly correlated with PFS (HR = 0.39, 95% CI = 0.16–0.94, *P* = 0.019). The presence of any mutation in cancer samples was significantly correlated with OS and PFS (HR = 0.38, 95% CI = 0.20–0.71, *P* = 0.002; and HR = 0.53, 95% CI = 0.30–0.92, *P* = 0.024, respectively). (Supplementary Table 3). Moreover, the concordance analysis between presence of the same mutation in both ctDNA and cancer sample showed that among the 20 samples with ctDNA presence, the concordance rate between ctDNA and tumour tissue was 35% (95% CI = 15.4–59.2). Unfortunately, statistical analyses could not be performed in this subgroup due to its too small size. Then, to determine whether ctDNA presence could be due to shedding from the cancer mass into the bloodstream, the kappa coefficient test showed a significant agreement between presence/absence of a mutation in ctDNA and cancer specimen (*P* = 0.0001).

### Small extracellular vesicles

**Fig. 3:**
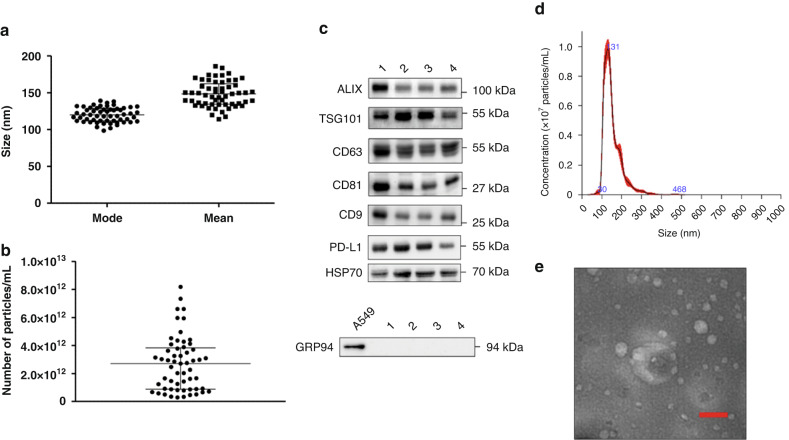
Characterization of sEVs isolated from plasma samples of 53 patients with NSCLC. **a** Mode and mean size of sEVs. **b** Mean concentration of sEVs in the 53 samples. **c** Representative western blots showing the expression of sEV markers (ALIX, TSG101, CD63, CD81, CD9), PD-L1 and HSP70 in lysates of sEVs isolated from four different plasma samples (upper panels). GRP94 was used as a negative control for cellular material contamination, and the total protein extract of the human lung cancer cell line A549 as a positive control for this contamination (lower panel). **d** Representative distribution of the size of particles isolated from plasma samples of patients with NSCLC obtained by nanoparticle tracking analysis. **e** Representative transmission electron microscopy image of the isolated sEVs. Scale bar, 100 nm; sEVs small Extracellular Vesicles.

The size distribution of sEVs isolated from 500 μl plasma samples from 53 patients was calculated using NTA (NS300). The particle mode and mean size values were 120.3 ± 9.9 and 148.2 ± 17.2 nm, respectively, confirming sEV isolation (Table 1). The mean concentration was 2.6 × 10^12^ particles/ml (Fig. 3a, b).

sEV enrichment was verified by transmission electron microscopy and western blotting using antibodies against ALIX, TSG101, CD63, CD81, CD9 (sEV markers), and also against GRP94 (negative control), HSP70 (cancer biomarker) [18], and PD-L1 (Fig. 3c–e).

PD-L1^+^ sEVs were detected in 88.5% of patients and their mean concentration was 16.9 ± 14.5 pg/ml. PD-L1^+^ sEV concentration was not associated with the total sEV concentration, CTC number, age, time since diagnosis, number of metastatic sites, number of previous systemic treatment lines, and total duration of systemic treatment (data not shown). However, PD-L1^+^ sEV concentration tended to decrease with longer intervals between diagnosis and inclusion (Spearman’s ρ = −0.26, *P* = 0.067). Total sEV concentration was significantly associated with the number of metastatic sites (Spearman’s ρ = 0.35, *P* = 0.011).

**Fig. 4:**
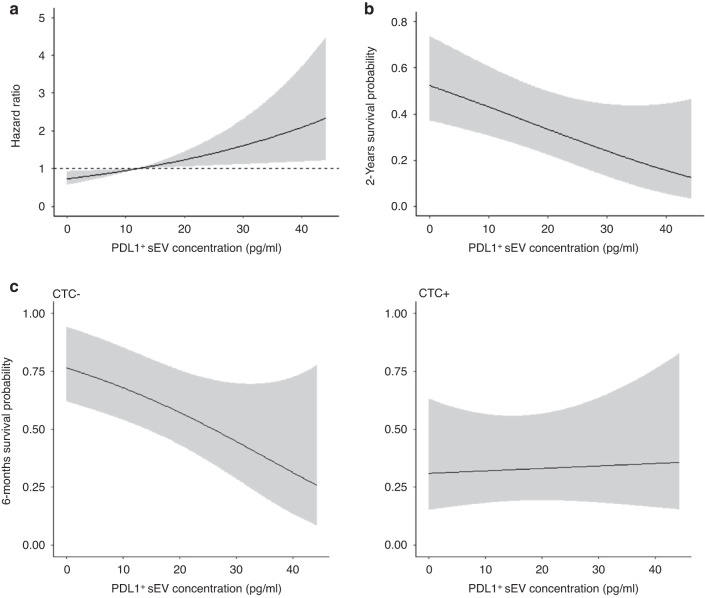
PD-L1^+^ sEV concentration and prognostic value. **a** PD-L1^+^ sEV concentration and overall survival (Hazard ratio). **b** 2-year survival probability in function of PD-L1^+^ sEV concentration, estimated from the univariable Cox model assuming linearity (*N* = 52 sample). **c** Six-month progression-free survival probability in function of PD-L1^+^ sEV concentration in patients with and without CTCs. Cox model that included PD-L1^+^ sEV concentration, CTC status, and their interaction. CTCs Circulating tumor cells.

Then, to assess their prognostic value (OS and PFS), PD-L1^+^ sEV and total sEV concentrations were used as continuous variables to obtain the most information and avoid using arbitrary cut-offs. The patient with PD-L1^+^ sEV concentration of 939 pg/ml was considered as an outlier and was excluded from the analysis (thus, *n* = 52 samples in total). In the univariable analysis, PD-L1^+^ sEV concentration was significantly associated with OS (*HR*_*increase of* 5 *pg*_ $=$ 1.14, 95% CI = 1.03–1.26, *P* = 0.016). To illustrate the effect of PD-L1^+^ sEV concentration on OS, the hazard ratio and 2-year survival rates were calculated in function of PD-L1^+^ sEV concentration and its median value from the univariable model (Fig. 4a, b). Higher PD-L1^+^ sEV concentration was associated with higher risk of death, and the survival probability decreased with higher PD-L1^+^ sEV concentrations. Conversely, total sEV concentration was not associated with OS (HR = 1.00, 95% CI = 0.93–1.08, *P* = 0.953). PD-L1^+^ sEV and total sEV concentrations were not correlated with PFS (HR = 1.08, 95% CI = 0.99–1.18, *P* = 0.095; HR = 0.99, 95% CI = 0.93–1.06, *P* = 0.813, respectively).

#### Interaction between PD-L1^+^ sEV concentration and CTC detection

51 patients were included in the model (*n* = 2 patients with missing values for sEVs or for CTC status). Comparison of the patients’ characteristics before and after the exclusion of these two patients did not highlight any significant difference (data was not shown). The interaction analysis did not find any significant interaction of PD-L1^+^ sEV and total sEV concentration with OS in function of CTC presence/absence (P interaction = 0.191 and 0.923, respectively) (Supplementary Table 4).

Conversely, PD-L1^+^ sEV concentration effect on PFS changed in function of CTC presence/absence (P interaction = 0.036). Specifically, higher PD-L1^+^ sEV concentration was significantly associated with poorer PFS in patients without CTCs (HR = 1.20, 95% CI 1.04–1.38, *P* = 0.011), but not in patients with CTCs (HR = 0.98, 95% CI 0.87–1.11, *P* = 0.809) (Supplementary Table 5). Analysis of 6-month PFS probability in function of PD-L1^+^ sEV concentration in patients with and without CTCs is shown below (Fig. 4c). The effect of total sEV concentration on PFS did not change in function of CTC status (P interaction = 0.710).

#### Multivariable analysis

The multivariable Cox model (*n* = 51 patients) included only PD-L1^+^ sEV concentration because total sEV concentration did not show any effect on survival in univariable analyses. After adjusting for CTC presence, number of previous systemic treatment lines, and histological type (squamous cell/basaloid carcinoma vs others), higher PD-L1^+^ sEV concentration remained associated with worse OS (HR = 1.15, 95% CI 1.04–1.28, *P* = 0.008), like CTC presence, systemic treatment lines and squamous cell/basaloid carcinoma histology (Supplementary Table 6). The different PD-L1^+^ sEV concentration effect on PFS in patients with and without CTCs was confirmed by the multivariable analysis (P interaction = 0.044) (Supplementary Table 7).

In patients without CTCs, high PD-L1^+^ sEV concentration was correlated with poorer PFS (HR = 1.20, 95% CI 1.05–1.39, *P* = 0.007), but not in patients with CTCs (*P* = 0.935) (Supplementary Fig. 2). Like for OS, previous systemic treatment lines and squamous cell/basaloid carcinoma also were associated with worse PFS (Supplementary Table 7).

### Combination of liquid biopsy markers and clinical outcome

To assess the prognostic impact of the combination of the three liquid biopsy biomarkers (ctDNA mutations, CTCs, PD-L1^+^ sEVs), first PD-L1^+^ sEV concentration cut-offs for OS (11.5 pg/ml) and PFS (8.6 pg/ml) were determined using the maximally selected rank statistics. The combination of CTC status, PD-L1^+^ sEV concentration, and ctDNA mutations that were previously correlated with OS and PFS in the 51 patients without missing data are described in Supplementary Table 8.

For each survival outcome (OS and PFS), a multivariable model that included the dichotomized PD-L1^+^ sEV concentration (high/low), CTC presence/absence, and ctDNA mutations was used to estimate the HR of each combination compared with the absence of other risk factors. For PFS, the interactions between the dichotomized PD-L1^+^ sEV concentration and CTC status were also added in the model. OS was worse in patients with CTCs only and high PD-L1^+^ sEV concentration (>11.5 pg/ml) only compared with patients without these risk factors (HR = 2.8, 95% CI = 1.46–5.39, *P* = 0.002; and HR = 2.73, 95% CI = 1.36–5.51, *P* = 0.005). OS was shorter also in patients with CTCs and at least one ctDNA mutation, and in patients with high PD-L1^+^ sEV concentration and at least one ctDNA mutation compared with patients without these risk factors (HR = 3.25, 95% CI = 1.38–7.65, *P* = 0.007; and HR = 3.17, 95% CI = 1.09–9.19, *P* = 0.033). These HR values were close to the HR values obtained for CTCs only and for high PD-L1^+^ sEV concentration only, suggesting that the addition of ctDNA mutations did not much influence the results. The worst HR value was observed in patients with all three risk factors (HR = 8.88, 95% CI = 2.79–28.31, *P* < 0.001), followed by patients with CTCs and high PD-L1^+^ sEV concentration (HR = 7.65, 95% CI = 3.11–18.83, *P* < 0.001) (Table 2).

**Table 2:** Hazard ratios (HR) for overall survival and progression-free survival in the presence of the different combinations of risk factors compared with patients without CTCs, low PD-L1^+^ sEV concentration and no ctDNA mutation (*N* = 51 patients with NSCLC without missing values for the three risk factors) (*N* = 51).

Risk factors	HR	95% CI	*P*-value
OS			
No risk factor	1.00	Ref	-
CtDNA mutation only	1.16	(0.61; 2.21)	0.649
CTCs only	2.8	(1.46; 5.39)	0.002
PD-L1^+^ sEV concentration >11.5 pg/ml only	2.73	(1.36; 5.51)	0.005
CTCs and PD-L1^+^ sEV concentration >11.5 pg/ml	7.65	(3.11; 18.83)	<0.001
CTCs and ctDNA mutation	3.25	(1.38; 7.65)	0.007
PD-L1^+^ sEV concentration >11.5 pg/ml and ctDNA mutation	3.17	(1.09; 9.19)	0.033
All the three risk factors	8.88	(2.79–28.31)	<0.001
PFS			
No risk factor	1.00	Ref	-
CtDNA mutation only	1.17	(0.64; 2.16)	0.607
CTCs only	4.89	(1.69; 14.2)	0.003
PD-L1^+^ sEV concentration >8.6 pg/ml only	4.23	(1.61; 11.1)	0.003
CTCs and PD-L1^+^ sEV concentration >8.6 pg/ml	6.1	(2.26; 16.46)	<0.001
CTCs and ctDNA mutation	5.74	(1.65; 19.95)	0.006
PD-L1^+^ sEV concentration >8.6 pg/ml and ctDNA mutation	4.96	(1.47; 16.78)	0.01
All the three risk factors	7.15	(2.04; 25.12)	0.002

Then, to determine whether the sequential addition of each risk factor decreased significantly survival, the same HRs were estimated using patients with CTCs only as the reference. The worst HR value for OS was observed in patients with CTCs and high PD-L1^+^ sEV concentration (HR = 2.73, 95% CI = 1.36–5.51, *P* = 0.005), suggesting a strong negative effect of the presence of both CTCs and high PD-L1^+^ sEV concentration. PFS was worse in patients with CTCs only (HR = 4.89, 95% CI = 1.69–14.2, *P* = 0.003) and with PD-L1^+^ sEV concentration >8.6 pg/ml only (HR = 4.23, 95% CI = 1.61–11.1, *P* = 0.003) compared with patients without these risk factors. On the other hand, PFS was comparable in patients with CTC and high PD-L1^+^ sEV concentration and in patient with only one of these risk factors (Table 2).

The 1-year and 2-year OS rates and the 6-month and 1-year PFS rates for each risk factor combination were estimated using the multivariable Cox model. In patients with CTCs or high PD-L1^+^ sEV concentration, both OS and PFS rates decreased significantly. (Supplementary Tables 9 and 10).

## Discussion

Liquid biopsy offers the opportunity to monitor cancer in the blood. Besides their predictive role, liquid biopsy markers could be an effective tool for the discovery of emerging resistance mechanisms, minimal residual disease monitoring, and early cancer detection. CTCs, ctDNA, and most recently EVs are fascinating complementary liquid biopsy analytes that can be employed in parallel in a variety of cancer care strategies [16].

In the last decade, the management of advanced NSCLC has improved (and consequently the OS and PFS rates) thanks to the identification of new candidate target genes [19, 20]. However, one of the main limitations to the widespread use of precision medicine in lung cancer is the difficult access to tumour tissue samples for accurate follow-up of disease progression and clonal adaptation. In this regard, liquid biopsy has attracted considerable attention. Many clinical trials have confirmed the clinical value of CTCs in patients with NSCLC (reviewed in detail [21]). Additionally, the expression of specific markers on CTCs can contribute to therapeutic decision-making and treatment response/resistance prediction. For instance, the evaluation of PD-L1 expression on CTCs from patients with NSCLC showed a dynamic increase in PD-L1^+^ CTCs that could be associated with resistance to immunotherapy [22]. Kloten et al. highlighted the use of CTCs as a diagnostic tool for PD-L1 expression analysis in patients with advanced NSCLC [23]. A previous report by our group [8] showed that PD-L1^+^ CTC detection is correlated with OS and PFS. However, studies in larger samples are needed to confirm this finding and to determine how PD-L1^+^ CTC detection might help to predict the response or resistance to anti-PD-1/PD-L1 therapies.

Furthermore, ctDNA mutation status can predict OS and PFS in patients with NSCLC undergoing treatment [24]. Interestingly, ctDNA collected at 1 month after chemoradiotherapy/radiotherapy initiation was optimal to predict the patients’ PFS and OS. Moreover, the dynamic change in ctDNA was closely associated with the clinical outcomes. This highlighted the possibility to adjust in real time treatment regimens in patients with inoperable localized NSCLC [25]. Moreover, ctDNA concentration has been associated with longer survival in patients with advanced NSCLC who received atezolizumab or docetaxel [26]. The most recent multi-centre randomized clinical trial aimed to evaluate the ascertain ctDNA response and define optimal timing and concordance with radiologic Response Evaluation Criteria in Solid Tumors (RECIST) response. Their funding demonstrated a sensitivity of ctDNA response for RECIST response of 82% (90% confidence interval (CI): 52–97%) and a specificity of 75% (90% CI: 56.5–88.5%) [27]. Interestingly, ctDNA concentration increased in patients who underwent chemotherapy, just 4 h after therapy, confirming the post-treatment ctDNA dynamic pattern [28]. In addition, ctDNA could be a biomarker for the early detection of molecular residual disease and for the prediction of postoperative relapse, thus facilitating the implementation of personalized adjuvant therapy at an early stage [29, 30]. However, ctDNA clinical value has been confirmed only in homogenous patients population, and has been mostly investigated in the pre- and post-treatment steps, unlike CTCs that behave independently of this factor [8].

Proteomic analysis of NSCLC samples showed that expression of lipopolysaccharide binding proteins in the sEV membrane allows differentiating between patients with metastatic and non-metastatic disease [31]. EV DNA displayed higher concordance with conventional tumour biopsies compared with ctDNA [32]. Different ALK-fusion variants were detected by digital PCR in EVs from plasma samples of patients with NSCLC [33]. A study found that for the assessment of clinical outcomes based on the detection of common BRAF, KRAS, and EGFR mutations, sEV nucleic acids are more sensitive that plasma ctDNA in patients with NSCLC [34]. PD-L1 levels in circulating sEVs give reliable information on PD-L1 expression in tumour biopsies. Monitoring circulating PD-L1^+^ sEVs may be useful to predict the tumour response to treatment and the clinical outcome [9].

In this proof of concept study, we investigated the prognostic value of different liquid biopsy biomarkers (CTCs, PD-L1^+^ CTCs, sEVs, PD-L1^+^ sEVs, and ctDNA) alone, and in combination, in a cohort of patients with NSCLC, regardless of cancer treatment, subtype, and stage, to determine whether their combination gives more precise prognostic information.

We showed that PFS and OS were worse in patients with PD-L1^+^ CTCs than in patients with PD-L1^−^ CTCs or without CTCs [8]. Moreover, we found that the concentration of PD-L1^+^ sEVs, evaluated as a continuous parameter (pg per ml), was associated with OS (HR = 1.14, 95% CI = 1.03–1.26, *P* = 0.016), but not with PFS (HR = 1.08, 95% CI = 0.99–1.18, *P* = 0.095). Similar results for OS were previously reported for melanoma [9]. Moreover, we observed that the PD-L1^+^ sEV concentration association with OS was not influenced by the patient’s CTC status. Conversely, higher PD-L1^+^ sEV concentration was significantly associated with poorer PFS in patients without CTCs. This suggests that the association of PD-L1^+^ sEVs and CTCs might allow stratifying patients more precisely (CTC^+^/ high PD-L1^+^ sEVs, CTC^+^/ low PD-L1^+^ sEVs, CTC^-^/high PD-L1^+^ sEVs, and CTC^−^/low PD-L1^+^ sEVs) for prognosis.

Total sEV concentration did not show any correlation with OS or PFS. This could be due to the heterogeneous origin (different cells and tissues) of blood EVs, as already shown by other studies [35]. Nevertheless, these results indicate that future works should mainly focus on PD-L1 expression variations in EVs.

In the present study, we also assessed ctDNA mutation status with the very sensitive and specific UltraSEEK™ method [36]. Several studies reported the clear benefits of assessing the presence of specific targetable mutations, such as *EGFR*, in ctDNA; however, currently this analysis is offered only to 15–25% of patients [37]. Here, we evaluated whether the analysis of a panel of NSCLC-specific mutations in ctDNA could predict survival, independently of treatment or molecular subtype. Although ctDNA is already used to guide treatment, especially targeted therapy [37], in our sample, ctDNA mutation detection did not have any predictive value, certainly, because the analysis was performed in the whole population, independently of the treatment type. This can be explained by the fact that unlike ctDNA that is released by dying tumour cells, CTCs and sEVs carry a complete functional “package” of cellular cargoes that can be exploited to better characterize each patient. Moreover, although ctDNA has become the predominant analyte for liquid biopsies to understand the cancer mutational landscape, it is not possible to determine whether the detected mutation is from tumour or dying/old cells. Moreover, ctDNA mutations could have a predictive value for the response to treatment (e.g. specific EGFR inhibitors for EGFR mutations or BRAF and MEK inhibitors) that could change the prognostic value. On the other hand, *KRAS* mutated tumours present an intrinsic bad prognosis that could be reversed by a good response to immunotherapy. ctDNA should not be analysed in terms of prognostic value on its own (presence/absence), but on the basis of the presence of specific mutations and or of its concentration. According to several studies, ctDNA concentration reflects the tumour burden and is associated with bad prognosis. However, our study was not designed to show such results. Additionally, some differences among studies could be due to the absence of a standardized method for ctDNA analysis. Interestingly, we observed a 35% of mutation concordance between ctDNA and tissue samples (95% CI = 15.4–59.2). Moreover, the kappa coefficient test reflected statistically significant agreement in presence/absence of mutations in ctDNA and tissue samples (*P* = 0.0001). This suggested that the detected ctDNA might be mutated DNA shed from the tumour mass into the bloodstream.

**Fig. 5:**
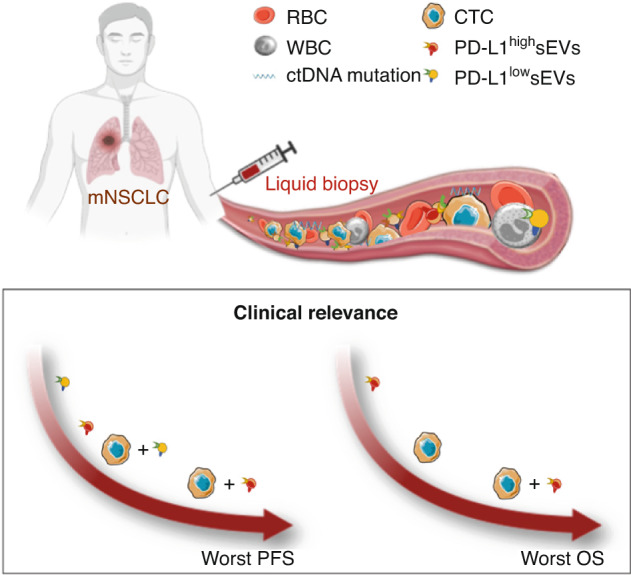
Schematic representation of the winning combination of liquid biopsy analytes to obtain prognostic information in patients with metastatic non-small cell lung cancer (mNSCLC). PFS Progression-free survival, OS Overall survival.

Lastly, we assessed the combination/complementarity of the three liquid biopsy analytes. We found that CTCs gave robust prognostic information that was independent of the molecular subtype and treatment [8]. The concentration of PD-L1^+^ sEVs, but not of total sEVs, brought prognostic information when combined with the CTC status. Indeed, PFS was shorter in patients without CTCs and high PD-L1^+^ sEV concentration. Moreover, the worst OS was observed in patients with high PD-L1^+^ sEV concentration and with CTCs (Fig. 5).

Currently, first-line immunotherapy is reserved to patients with NSCLC and PD-L1 expression level >50%. Moreover, PD-L1 level seems to be the best predictor of the response to immunotherapy [38–40]. However, immunotherapy response rates vary between 15 and 45% in patients selected on the basis of PD-L1 expression [41], and some patients who do not express PD-L1 might respond to immunotherapy [41, 42]. These observations can be explained by the intra- and inter-tumoral heterogeneity in PD-L1 expression [43, 44]. In addition, PD-L1 expression analysis is not always feasible in the initial sample due to the insufficient quantity of tumour material and the difficulties to perform a new biopsy. Therefore, PD-L1 analysis in liquid biopsy analytes seems to be a promising approach because it may better reflect tumour heterogeneity compared with the tissue biopsy and also due to its non-invasive nature. We previously showed that PD-L1^+^ CTCs were significantly correlated with OS and PFS and that prognosis is worse in patients with PD-L1^+^ CTCs than with PD-L1^-^ CTCs [8]. However, here, the PD-L1^+^ CTC subgroup was not included as a risk factor in the combinatory analysis, due to the small number of patients (*N* = 5). As previous and current results confirmed the importance of PD-L1 expression in CTCs and sEVs, we think that assessing PD-L1 expression in CTCs and sEVs can provide reliable information specifically in patients receiving immunotherapy. An ongoing clinical trial (NCT04025541) in patients with NSCLC receiving immunotherapy evaluates the importance of CTCs, sEVs, ctDNA, and immune cell changes and their combined analysis for predicting the response to tumour-related events (e.g. treatment, surgery).

We must acknowledge some limitations. Despite the fact that liquid biopsy is a powerful approach in oncology, it has advantages and limitations. Indeed, technical inconsistencies and lack of standardization hinder its broad and routine use in the clinic. For instance, ctDNA analysis is an attractive approach due to its simplicity, but it is limited to the analysis of DNA-related abnormalities. Mutations can be detected in ctDNA and are increasingly used to predict the response to targeted therapies; however, longitudinal monitoring of ctDNA mutations is needed to track emergent therapy resistance. CTC analysis offers cell-wide characterization; nonetheless, it is challenging to identify a population of rare cells. There is no standardized approach for sEV isolation, and it is impossible to isolate a pure sEV population. These limitations concern also our study. Moreover, due to the limited number of patients in this cohort, analysis of subgroups with different combinations of the three predictive biomarkers was not possible. Furthermore, the CellSearch® system, the only FDA-approved system in the USA and the gold standard for CTC detection, was used for this study. However, it is well known that in patients with NSCLC, many CTCs do not have sufficient epithelial characteristics, and therefore might escape detection. Also, there are different techniques for isolating and detecting ctDNA and EVs, and it can be difficult to determine which approach is the most effective. Thus, our findings must be interpreted with caution due to the lack of standardized procedures for the analysis of liquid biopsy analytes. This highlights the urgent need for a multidimensional effort to optimize and standardize accessible and efficient methods.

## Conclusion

This study showed that CTC presence and high PD-L1^+^ sEV concentration are predictive biomarkers on their own and that their combination predicts worse prognosis. Future clinical trials should focus on combining the analysis of different liquid biopsy analytes for the personalized management of all patients. Indeed, all different biomarkers must be considered together to have a better view for personalized medicine and targeted therapeutic approaches. Moreover, more interventional clinical trials with higher number of patients are required to confirm our findings and to determine whether this combination of liquid biopsy biomarkers helps to predict prognosis. For this purpose, international consortia including partners from academia and industry, such as the European Liquid Biopsy Society (ELBS), have been established to standardize and organize multicentre clinical trials. Finally, this new multi-omics liquid biopsy approach can lead to the development of an algorithm that can combine data from different liquid biopsy biomarkers to obtain precise tumour information for guiding therapeutic decision-making.

### Supplementary information

Supplemental file revision

## Data Availability

All data analysed during this study are included in this published article.
