# Supplementary material for: Circulating tumour cells and PD-L1-positive small extracellular vesicles: the liquid biopsy combination for prognostic information in patients with metastatic non-small cell lung cancer
Source: Br J Cancer. 2023 Nov 16;130(1):63–72. doi: 10.1038/s41416-023-02491-9 (PMC10781977; doi:10.1038/s41416-023-02491-9)
Supplement: Supplementary file 1 — Supplemental file revision [file 41416_2023_2491_MOESM1_ESM.docx]

**Supplemental data**

| **A)**  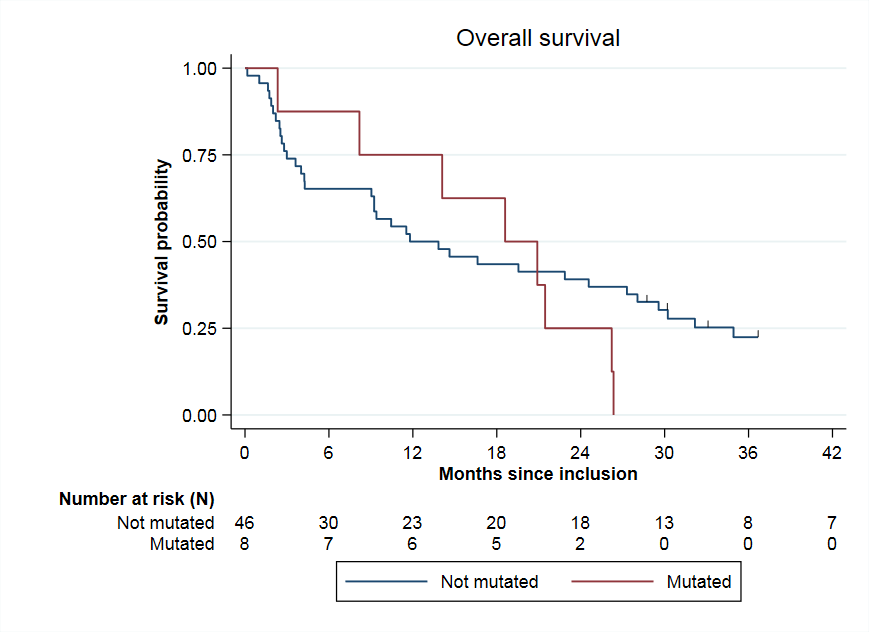  P=0.407 | **B)**  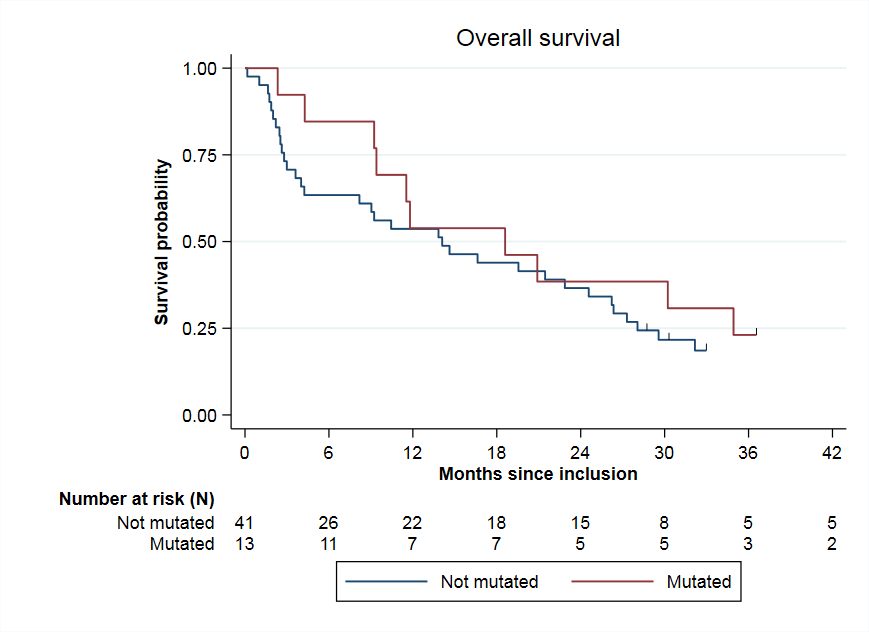  P=0.524 |
| --- | --- |
| **C)**  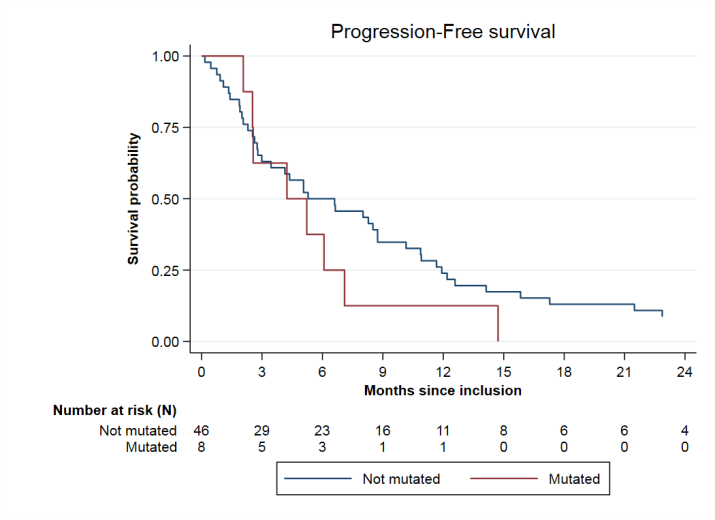  P=0.308 | **D)**  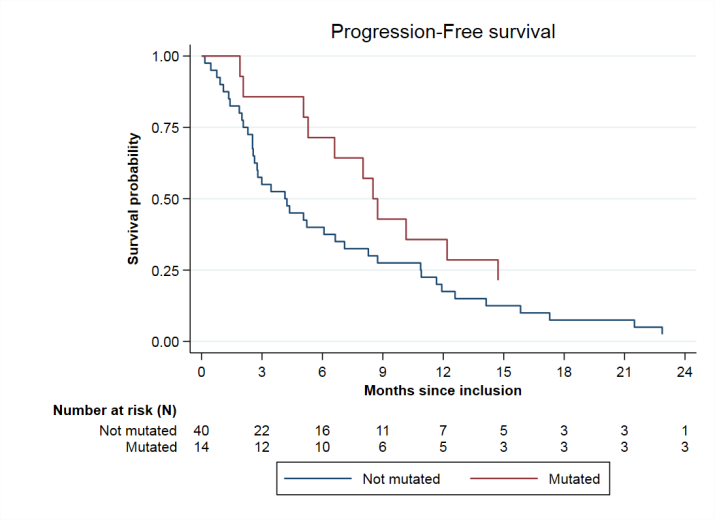  P=0.488 |
| **Supplemental Figure 1:** Correlation of *KRAS* and *EGFR* mutations in ctDNA with overall survival and progression-free survival. **A)** Kaplan-Meier curve for overall survival according to the *KRAS* mutation status. **B)** Kaplan-Meier curve for overall survival according to the *EGFR* mutation status. **C)** Kaplan-Meier curve for progression-free survival according to the *KRAS* mutation status. **D)** Kaplan-Meier curve for progression-free survival according to the *EGFR* mutation status. | |

| **** |
| --- |
| **Supplemental Figure 2:** Six-month progression-free survival probability in function of PD-L1^+^ sEV concentration in patients with and without CTCs, who had one or two previous lines of systemic chemotherapy, and who did not have a squamous cell or basaloid carcinoma (estimations from the multivariable model presented in **Table 2**). |

| **Supplemental Table 1**: Univariable analysis of overall survival (OS) and progression-free survival (PFS) in patients with advanced NSCLC (N = 54). |
| --- |
| \| OS (N=54) \| \| \| \| \| \| --- \| --- \| --- \| --- \| --- \| \| Variables \| **Number of events / N** \| **HR** \| **CI 95%** \| **P-value** \| \| CTC presence \| \| \| \| **P <0.001** \| \| No \| 22/30 \| 1.00 \| Ref \| \| Yes \| 22/23 \| 3.06 \| [1.65; 5.70] \| \| NA \| 1/1 \|  \|  \| \| PD-L1^+^ CTC presence \| \| \| \| **P = 0.097** \| \| No \| 39/48 \| 1.00 \| Ref \| \| Yes \| 5/5 \| 2.45 \| [0.95; 6.36] \| \| NA \| 1/1 \|  \|  \| \| PD-L1 status in CTCs \| \| \| \| **P = 0.002** \| \| CTC^-^ \| 22/30 \| 1.00 \| Ref \| \| PD-L1^-^ CTC \| 17/18 \| 2.93 \| [1.51; 5.68] \| \| PD-L1^+^ CTC \| 5/5 \| 3.63 \| [1.33; 9.91] \| \| NA \| 1/1 \|  \|  \| \|  \| \| \| \| \| |
| \| PFS (N=54) \| \| \| \| \| \| --- \| --- \| --- \| --- \| --- \| \| Variable \| **Number of events / N** \| **HR** \| **CI 95%** \| **P-value** \| \| CTC presence \| \| \| \| **P = 0.006** \| \| No \| 28/30 \| 1.00 \| Ref \| \| Yes \| 23/23 \| 2.27 \| [1.28; 4.04] \| \| NA \| 1/1 \|  \|  \| \| PD-L1^+^ CTC presence \| \| \| \| **P = 0.442** \| \| No \| 46/48 \| 1.00 \| Ref \| \| Yes \| 5/5 \| 1.47 \| [0.58; 3.74] \| \| NA \| 1/1 \|  \|  \| \| PD-L1 status in CTCs \| \| \| \| **P = 0.020** \| \| CTC^-^ \| 28/30 \| 1.00 \| Ref \| \| PD-L1^-^ CTC \| 18/18 \| 2.43 \| [1.30; 4.55] \| \| PD-L1^+^ CTC \| 5/5 \| 1.89 \| [0.72; 4.95] \| \| NA \| 1/1 \|  \|  \| |

| **Supplemental Table 2:** Baseline characteristics in function of the presence/absence of ctDNA mutation(s) |
| --- |
| \| Variable \| No ctDNA mutation  N=34 \| At least one ctDNA mutation  N=20 \| Test \| \| --- \| --- \| --- \| --- \| \| Age \| \| \| Kruskal-Wallis **P=0.603** \| \| N \| 34 \| 20 \| \| Mean (SD) \| 63.7 (12.8) \| 65.8 (10.2) \| \| Median (Q1 ; Q3) \| 67.0 (53.0;74.0) \| 67.0 (56.5; 75.0) \| \| Missing \| 0 \| 0 \| \| Sex \| \| \| Fisher’s Exact **P=1.000** \| \| Man \| 19 (55.9%) \| 12 (60.0%) \| \| Woman \| 15 (44.1%) \| 8 (40.0%) \| \| Smoking \| \| \| Fisher’s Exact **P=0.736** \| \| Never smoker \| 5 (15.2%) \| 2 (10.5%) \| \| Previous smoker \| 22 (66.7%) \| 15 (79%) \| \| Current smoker \| 6 (18.1%) \| 2 (10.5%) \| \| Missing \| 1 \| 1 \| \| BMI (kg/m²) \| \| \| Kruskal-Wallis **P=0.186** \| \| N \| 32 \| 18 \| \| Mean (SD) \| 23.2(4.2) \| 24.8 (4.0) \| \| Median (Q1; Q3) \| 22.8 (19.5; 26.0) \| 24.2 (21.6; 26.7) \| \| Missing \| 2 \| 2 \| \| Medical history of disease \| \| \| Fisher’s Exact **P=0.510** \| \| No \| 8 (23.5%) \| 3 (15.0%) \| \| Yes \| 26 (76.5%) \| 17 (85.0%) \| \| Time since cancer diagnosis (months) \| \| \| Kruskal-Wallis **P=0.210** \| \| N \| 34 \| 20 \| \| Mean (SD) \| 15.5 (18.5) \| 23.2 (25.9) \| \| Median (Q1; Q3) \| 8.3 (4.6;20.7) \| 13.1 (6.1; 29.1) \| \| Missing \| 0 \| 0 \| \| Histological type \| \| \| Fisher’s Exact **P=0.717** \| \| Adenocarcinoma \| 23 (67.6%) \| 16 (80.0%) \| \| Squamous cell/basaloid carcinoma \| 8 (23.5%) \| 3 (15.0%) \| \| Other types \| 3 (8.8%) \| 1 (5.0%) \| \| cT \| \| \| Fisher’s Exact **P=0.378** \| \| 1-2 \| 4 (15.4%) \| 6 (35.3%) \| \| 3 \| 9 (34.6%) \| 5 (29.4%) \| \| 4 \| 13 (50.0%) \| 6 (35.3%) \| \| Missing \| 8 \| 3 \| \| cN \| \| \| Fisher’s Exact **P=0.397** \| \| 0 \| 10 (32.3%) \| 9 (50.0%) \| \| 1-2 \| 10 (32.3%) \| 3 (16.7%) \| \| 3 \| 11 (35.4%) \| 6 (33.3%) \| \| Missing \| 3 \| 2 \| \| cM \| \| \| Fisher Exact **P =0.608** \| \| 0 \| 3 (8.8%) \| 0 (0.0%) \| \| 1a \| 7 (20.6%) \| 4 (20.0%) \| \| 1b-1c \| 24 (70.6%) \| 16 (80.0%) \| \| Number of metastatic sites \| \| \| Kruskal-Wallis **P=0.178** \| \| N \| 34 \| 20 \| \| Mean (SD) \| 2.3 (1.6) \| 2.9 (1.4) \| \| Median (Q1; Q3) \| 2.0 (1.0;3.0) \| 3.0 (2.0; 4.0) \| \| Missing \| 0 \| 0 \| |

| **Supplemental Table 3**: Hazard ratio for mutation status in tissue on Overall survival (OS) and Progression-Free survival (PFS). Cox model using data from the patients with NSCLC included in the study (N=54). |
| --- |
| \| OS (N=54) \| \| \| \| \| --- \| --- \| --- \| --- \| \|  \| **Univariate** \| \| \| \|  \| **Number of events / N** \| **HR** \| **IC 95%** \| \|  \|  \|  \|  \| \| KRAS mutation (tissue) \|  \|  \| P=0.211 \| \| Not mutated \| 35/41 \| 1.00 \| Ref \| \| Mutated \| 10/13 \| 0.65 \| [0.32 ; 1.31] \| \|  \|  \|  \|  \| \| EGFR mutation (tissue) \|  \|  \| P=0.168 \| \| Not mutated \| 40/47 \| 1.00 \| Ref \| \| Mutated \| 5/7 \| 0.54 \| [0.21 ; 1.39] \| \|  \|  \|  \|  \| \| Any mutation in tissue \|  \|  \| P=0.002 \| \| Not mutated \| 29/31 \| 1.00 \| Ref \| \| Mutated \| 16/23 \| 0.38 \| [0.20 ; 0.71] \| \|  \| \| \| \| \| PFS (N=54) \| \| \| \| \|  \| **Univariate** \| \| \| \|  \| **Number of events / N** \| **HR** \| **IC 95%** \| \|  \|  \|  \|  \| \| KRAS mutation (tissue) \|  \|  \| P=0.677 \| \| Not mutated \| 39/41 \| 1.00 \| Ref \| \| Mutated \| 13/13 \| 0.87 \| [0.46 ; 1.65] \| \|  \|  \|  \|  \| \| EGFR mutation (tissue) \|  \|  \| P=0.019 \| \| Not mutated \| 46/47 \| 1.00 \| Ref \| \| Mutated \| 6/7 \| 0.39 \| [0.16 ; 0.94] \| \|  \|  \|  \|  \| \| Any mutation in tissue \|  \|  \| P=0.024 \| \| Not mutated \| 30/31 \| 1.00 \| Ref \| \| Mutated \| 22/23 \| 0.53 \| [0.30 ; 0.92] \| |

| **Supplemental Table 4**: Evaluation of potential interactions between PD-L1^+^ sEV and total sEV concentration and CTC status and their effect on overall survival. Cox model using data from the patients with NSCLC included in the study (N=53). |
| --- |
| \|  \| CTC status \| \| \| \|  \| \| --- \| --- \| --- \| --- \| --- \| --- \| \|  \| **CTC^-^ (N=30)** \| \| **CTC^+^ (N=23)** \| \| **P-value Interaction** \| \| Variable \| **Coefficient** \| **95% CI** \| **Coefficient** \| **95% CI** \|  \| \| PD-L1^+^ sEV concentration \|  \| **P=0.010** \|  \| **P=0.150** \| **P=0.191** \| \| 5 pg/ml increase \| 1.23 \| [1.05; 1.46] \| 1.09 \| [0.97; 1.22] \|  \| \| sEV concentration \|  \| **P=0.827** \|  \| **P=0.724** \| **P=0.923** \| \| 500 x 10^9^ sEVs/ml increase \| 0.99 \| [0.89; 1.10] \| 0.98 \| [0.88; 1.09] \|  \| |

| **Supplemental Table 5**: Evaluation of potential interactions between PD-L1^+^ sEV and total sEV concentration and CTC status and their effect on progression-free survival. Cox model using data from the patients with NSCLC included in the study (N=53). |
| --- |
| \|  \| CTC status \| \| \| \|  \| \| --- \| --- \| --- \| --- \| --- \| --- \| \|  \| **CTC^-^ (N=30)** \| \| **CTC^+^ (N=23)** \| \| **P-value Interaction** \| \| Variable \| **Coefficient** \| **95% CI** \| **Coefficient** \| **95% CI** \|  \| \| PD-L1^+^ sEV concentration \|  \| **P=0.011** \|  \| **P=0.809** \| **P=0.036** \| \| 5 pg/ml increase \| 1.20 \| [1.04; 1.38] \| 0.98 \| [0.87; 1.11] \|  \| \| sEV concentration \|  \| **P=0.602** \|  \| **P=0.942** \| **P=0.710** \| \| 500 x 10^9^ sEVs/ml increase \| 0.97 \| [0.90; 1.07] \| 1.00 \| [0.90; 1.13] \|  \| |

| **Supplemental Table 6:** Adjusted hazards ratios (HR) for the variables in the multivariable Cox model for overall survival (N=51). |
| --- |
| \| Variable \| HR \| 95% CI \| \| --- \| --- \| --- \| \| PD-L1^+^ sEV concentration \|  \| **P=0.008** \| \| 5 pg/ml increase \| 1.15 \| [1.04; 1.28] \| \| Presence of CTCs \|  \| **P<0.001** \| \| No \| 1.00 \| Ref \| \| Yes \| 4.76 \| [2.20; 10.26] \| \| Number of previous treatment lines \|  \| **P=0.026** \| \| 0 \| 1.00 \| Ref \| \| 1-2 \| 2.48 \| [1.16; 5.25] \| \| >2 \| 4.33 \| [0.93; 8.21] \| \| Squamous cell/basaloid carcinoma \|  \| **P=0.003** \| \| No \| 1.00 \| Ref \| \| Yes \| 3.80 \| [1.70; 8.45] \| |

| **Supplemental Table 7:** Adjusted hazards ratios (HR) in the multivariable Cox model for progression-free survival (N=51). |
| --- |
| \| Variable \| HR \| 95% CI \| \| --- \| --- \| --- \| \| PD-L1^+^ sEV concentration* \|  \|  \| \| *Patients without CTCs* \|  \| **P=0.007** \| \| 5 pg/ml increase \| 1.20 \| [1.05; 1.39] \| \| *Patients with CTCs* \|  \| **P=0.935** \| \| 5 pg/ml increase \| 0.99 \| [0.87; 1.13] \| \| Number of previous treatment lines \|  \| **P=0.047** \| \| 0 \| 1.00 \| Ref \| \| 1-2 \| 2.31 \| [1.15; 4.66] \| \| >2 \| 1.86 \| [0.65; 5.33] \| \| Squamous cell/basaloid carcinoma \|  \| **P=0.019** \| \| No \| 1.00 \| Ref \| \| Yes \| 2.90 \| [1.25; 6.72] \| \| *P-value interaction= 0.044 \| \| \| |

| **Supplemental Table 8:** Number (percentage) of patients with the indicated marker combinations (CTC, PD-L1^+^ sEV concentration, and ctDNA mutations) used for the combination analysis for overall survival (OS) and progression-free survival (PFS). |
| --- |
| \| OS (N=51) \| \| \| \| --- \| --- \| --- \| \| Risk factors \| **Number** \| **Percentage** \| \| No CTC, no ctDNA mutation and low PD-L1^+^ sEV concentration (i.e. <11.5 pg/ml) \| 8 \| (15.7%) \| \| CTCs only \| 4 \| (7.8%) \| \| CtDNA mutation only \| 7 \| (13.7%) \| \| High PD-L1^+^ sEV concentration only (i.e. >11.5 pg/ml) \| 10 \| (19.6%) \| \| CTCs and ctDNA mutation \| 5 \| (9.8%) \| \| CTCs and high PD-L1^+^ sEV concentration \| 9 \| (17.6%) \| \| High PD-L1^+^ sEV concentration and ctDNA mutation \| 3 \| (5.9%) \| \| All three risk factors \| 5 \| (9.8%) \| \| Number of risk factors \| \| \| \| 0 \| 8 \| (15.7%) \| \| 1 \| 21 \| (41.2%) \| \| 2 \| 17 \| (33.3%) \| \| 3 \| 5 \| (9.8%) \| \|  \| \| \| |

| PFS (N=51) | | |
| --- | --- | --- |
| Risk factors | **Number** | **Percentage** |
| No CTCs, no ctDNA mutation, and low PD-L1^+^ sEV concentration (i.e. <8.6 pg/ml) | 6 | (11.8%) |
| CTCs only | 4 | (7.8%) |
| CtDNA mutation only | 4 | (7.8%) |
| High PD-L1^+^ sEV concentration only (i.e. >8.6 pg/ml) | 12 | (23.5%) |
| CTCs and ctDNA mutation | 5 | (9.8%) |
| CTCs and high PD-L1^+^ sEV concentration | 9 | (17.6%) |
| High PD-L1^+^ sEV concentration and ctDNA mutation | 6 | (11.8%) |
| All three risk factors | 5 | (9.8%) |
| Number of risk factors | | |
| 0 | 6 | (11.8%) |
| 1 | 20 | (39.2%) |
| 2 | 20 | (39.2%) |
| 3 | 5 | (9.8%) |

| **Supplemental Table 9**: Overall survival rates associated with the different combinations of risk factors (N=51 patients with NSCLC without missing values for the three risk factors). |
| --- |
| \| Risk factors \| 1-year survival (%) \| 95% CI \| 2-year survival (%) \| 95% CI \| \| --- \| --- \| --- \| --- \| --- \| \| No risk factor \| 82 \| (69.8; 96.5) \| 69.5 \| (52.8; 91.4) \| \| CtDNA mutation only \| 79.5 \| (66.5; 95) \| 65.6 \| (48.4; 88.8) \| \| CTCs only \| 57.4 \| (36.2; 91.1) \| 36.1 \| (16; 81.3) \| \| PD-L1^+^ sEV concentration >11.5 pg/ml only \| 58.2 \| (40.8; 83) \| 37 \| (20; 68.4) \| \| CTCs and PD-L1^+^ sEV concentration >11.5 pg/ml \| 22 \| (9; 53.5) \| 6.2 \| (1.2; 31.3) \| \| CTCs and ctDNA mutation \| 52.5 \| (33.1; 83.4) \| 30.6 \| (13.6; 69.2) \| \| PD-L1^+^ sEV concentration >11.5 pg/ml and ctDNA mutation \| 53.3 \| (32.9; 86.5) \| 31.5 \| (13.3; 74.7) \| \| All three risk factors \| 17.2 \| (5.7; 52.2) \| 3.9 \| (0.5; 31.1) \| |

| **Supplemental Table 10:** Progression-free survival rates associated with the different combinations of risk factors (N=51 patients with NSCLC without missing values for the three risk factors). |
| --- |
| \| Risk factors \| 6-month survival (%) \| 95% CI \| 1-year survival (%) \| 95% CI \| \| --- \| --- \| --- \| --- \| --- \| \| No risk factor \| 83.3 \| (69.3; 100) \| 65.3 \| (44; 96.8) \| \| CtDNA mutation only \| 80.7 \| (66.2; 98.4) \| 60.6 \| (38.9; 94.4) \| \| CTC^+^ only \| 40.9 \| (20.1; 83.5) \| 12.4 \| (2.5; 62.9) \| \| PD-L1^+^ sEV concentration >8.6 pg/ml only \| 46.2 \| (29; 73.4) \| 16.5 \| (6.1; 44.2) \| \| CTCs and PD-L1^+^ sEV concentration >8.6 pg/ml \| 32.8 \| (16; 67.4) \| 7.4 \| (1.5; 37) \| \| CTCs and ctDNA mutation \| 35.1 \| (16.4; 75) \| 8.7 \| (1.3; 55.8) \| \| PPD-L1^+^ sEV concentration >8.6 pg/ml and ctDNA mutation \| 40.4 \| (23; 70.9) \| 12 \| (3.1; 46.8) \| \| All three risk factors \| 27.1 \| (10.9; 67) \| 4.7 \| (0.5; 43.6) \| |
